# Clinic Atlanta Medical College

**Published:** 1876-04

**Authors:** A. W. Calhoun

**Affiliations:** Professor of Diseases of the Eye and Ear


### Clinic Atlanta Medical College.

A. W. CALHOUN, M.D., Professor of Diseases of the Eye and Ear.

(*Continued from page* 744 *of March Journal.*)

CASE XXXIX.—*Ciliary Neuralgia of two months' duration, cured by Quinine.*—L. W., æt. thirty-two, Atlanta, Ga. Aside from some menstrual derangement, the patient is in good general health. About two months since, pain began to occur in both balls in the region of the ciliary bodies, beginning at noon of each day and continuing until late at night. During the attacks the pain becomes so severe, and produces such intolerance of light, that she is obliged to confine herself very frequently to a darkened room, in order to get a little relief. The pain is not only in the ball, although severest there, but extends to the supra-orbital region and down alongside the nose. The conjunctiva becomes injected, in a circular form, around the cornea, and a rather excessive amount of tears is secreted. During the forenoon the eye is in perfect condition, giving no uneasiness whatever until towards noon, when the pain, as before described, begins. Interiorly nothing unusual was discovered. The patient had not been exposed to any malarious influence, yet the case was clearly one of periodic ciliary nervous irritation.

She was put upon quinine, three grains, three times daily, with ten drops of Fowler's solution. In addition, small portions of calomel were given every night or two, and strong hot mustard foot baths were used just before the expected approach of each attack.

At her next visit, one week later, she reported not having had an attack for the two or three preceding days. Saw her again after one month, and there was still no return of the disease.

Case XL.—*Injury to one Eye resulting in Sympathetic Inflammation and consequent Blindness in the other.—Iridectomy.* D. T., æt. twenty-six, Elberton, Ga., was blown up while blasting rock some two years ago, producing such injury to the right eye that, after much inflammation, it collapsed (phthisis bulbi.) The left cornea was also much injured, but this eye recovered, leaving him sufficient vision to attend to his work. One month after the injury, however, the left eye began to fail by reason of a slow sub-acute inflammation (sympathetic inflammation) extending from the injured (and not yet recovered) eye along the ciliary nerves. This continued, with considerable pain in both eyes, until the vision also of the left was so reduced that there was only a good perception of light remaining. Upon his presenting himself for treatment, the right eye was found shrunken, and the left with its iris changed in color and the pupil filled with a thick white membrane, not only filling the pupilary space, but even extending through the pupil into the anterior chamber. There was very good perception of light in the left eye, he being able to distinguish at once light from darkness when exposed to the one or the other.

Sympathetic inflammation usually destroys the vitality (so to speak) of the choroid, iris and ciliary bodies; but the ability to perceive light was so acute that it was determined to make a large iridectomy, and thereby, if possible, to allow the entrance of more light into the interior of the eye.

Accordingly, the operation was performed, making a large artificial pupil, but it was immediately seen that the result would be negative, inasmuch as the anterior surface of the lens was completely covered with the same exudative membrane which was seen in the pupilary space. This state of things is the same as usually found after sympathetic inflammation, so that except in the very beginning of the disease (and then often without avail) an iridectomy amounts to nothing. Had the collapsed ball been removed and the iridectomy made when the inflammation first began in the left eye, doubtless the vision here would have been retained.

It is now evident in this case that the deeper structures have become irrevocably impaired from the inflammation, and further interference, such as another iridectomy and the removal of the lens, was not thought advisable. Recovered in a few days, and dismissed.

Case XLI.—*Suppurative Inflammation (Otorrhœa) of the Middle Ear, resulting in Necrosis of the Mastoid Process.—Removal of a large portion of the Necrosed Mastoid.*—O. P., æt. twenty-two, Charlotte, N. C., has had for more than a year a profuse and fœtid discharge, commonly called otorrhœa, from the middle ear of the left side, evidently the result of syphilis, just now showing itself in its secondary form. The drum-head is almost totally destroyed, leaving only a portion of its very periphery, and the only part of the ossicles to be seen is a small piece of the body of the malleus, the other bones having been destroyed by the suppurative inflammation. The hearing power is entirely gone, the ticking of the watch not being perceived, even when pressed against the ear. The region of the mastoid process is very much swollen, hard and painful, and the auricle is forced off from the head and stands much lower than the right. Just above the ear, in the temple, is a small opening, from which pus is constantly oozing, drop by drop. A probe introduced into this opening passes down towards the mastoid, and strikes upon diseased bone.

A long incision was made, of about one and a half inches in length, over and down upon the mastoid bone, letting out a small amount of pus. It was necessary to make the cut fully from one half to three quarters of an inch deep before reaching the bone, on account of the excessive thickening of the parts. In diseases of this kind, this thickening is the rule, and one should not hesitate to cut just as deep as may be necessary, in order to find the bone, and not unfrequently the incision will have to be made half an inch or even more in depth. Blood flowed out through the meatus auditorius externus, thus showing the diseased connection between the mastoid process and the middle ear. Necrosed bone could be readily detected at the bottom of the incision, but it being difficult to detach it, the wound was packed with lint, and the patient directed to return on the following day, thus giving time for the dead bone to loosen itself. Upon his return, a piece of the process of the size of the end of the fore-finger was removed, and two days later another similarly sized portion was taken away. The wound was kept open, and milk warm salt water thrown in upon the mastoid surface, washing it off and passing out through the external meatus.

He was put upon iodide potassium and bi-chloride mercury, in full doses, and a solution containing—

| | |
|---|---|
| Tinct. myrrh, . . . . . . . | ℥ss. |
| Zinci. sulph., . . . . . . . | gr. v. |
| Morphiæ sulph., . . . . . . . | gr. ij. |
| Aquæ, distil., . . . . . . | ℥ss. |

was poured (milk warm) into the canal and middle ear several times daily, after washing the parts out by means of the syringe with warm salt water.

The patient left for his home before being entirely cured, but up to the time of his leaving the wound had very nearly healed, the swelling had mostly subsided, and the auricle was assuming its natural appearance and position. The discharge from the middle ear had very materially reduced, and there was no reason to doubt but that this also will entirely cease.

In all cases of mastoid complication, whether the bone has as yet become diseased or not, a free incision over it should be made, so as to prevent many dangerous consequences which might result from it.

CASE XLII.—*Traumatic Cataract.—Extraction, with consequent Iritis and Secondary Cataract.—A subsequent Iridectomy, with Complete Success.*—L. R., æt. thirty-one, Zebulon, Ga., was blown up five years ago, while blasting rock in a well, resulting in the entire destruction (phthisis bulbi) of the left ball and the formation of cataract in the right, due to the severe inflammation which followed in this eye and the concussion at the time of the explosion. There was evidently a violent iritis, for there now exists two or three extensive posterior synechiæ, binding the pupillary margin down to the anterior capsule of the lens, allowing only a very slight dilatation of the pupil under the use of atropine.

The cataract was removed by Graefe's linear extraction, and the usual test of vision applied, viz, holding the fingers before the eye, which were counted without the least difficulty. On the second day afterwards iritis ensued, and notwithstanding the application of the most active remedies for its control, it continued, and the natural and artificial (iridectomy) pupils were totally closed by a thick exudative membrane (secondary cataract). As soon as practicable the patient was dismissed, with directions to return as soon as the eye was entirely free from soreness and redness.

Two months later he returned, and the eye being in good condition, another operation was made, viz, *a large outward iridectomy*, taking the precaution at the same time to cut and break into many pieces the membrane in the pupil and behind the iris. Immediately he could see as distinctly as is possible in such cases.

The wound healed, and in ten days he was dismissed, with vision equal to 20-100 with cataract glass No. 3¾, and could read Snellen No. 4½ with cataract glass No. 2½. The vision was tested sooner than usual after such operations, and hence the full success could not as yet be seen. Without doubt, the sight will be doubly increased in the course of six or eight weeks.

### Notes Taken from Lectures of A.W. Calhoun, M.D., Delivered in Atlanta Medical College, January, 1876, for Special Instruction of Medical Class.

By W. S. KENDRICK, M.D.

*Cataract.*—An opacity of the lens; lens behind iris; double convex; posterior surface more convex, surrounded by capsule, anterior and posterior; both connected and one, held by suspensary ligament; suspensary ligament, extension forward of membrane from ora serrata; lens composed of concentric layers, nucleus in centre; cataract, disease and opacity of lens; if lens transparent no cataract. Two varieties, hard and soft; can usually tell them by appearance; hard after 25th year, usually soft under that age; exceptions to both. Hard, amber color, deep and light; some whitish or grayish; always contain some amber; amber color comes from nucleus. Several varieties, amongst which is black cataract, dark amber, not really black.

Soft cataract in young, looks soft, milky appearance, chalky. Lamellar, or zonular, begins in nucleus; nucleus opaque; periphery clear and transparent. In young often not congenital; often result of convulsions; lens, in children, does not fit closely in capsule; shaken by convulsions; traumatic.

Cortical cataract, cortical substance opaque; can see transparent portion under cortical substance; opaque at periphery. This species of cataract may be divided into anterior and posterior cortical; shows itself in radii running out; increases slowly; may be years before patient goes blind. Cortical mostly in old; zonular often in young. Anterior capsular cataract often in infants; small white spot on the capsule in pupil; some born this way, or result of inflammation. Child *in utero;* subject to inflammation; deposit on anterior capsule; result of inflammation *in utero;* often have suppuration and perforation of cornea.

Diagnosis of cataract simple and easy. Be guarded in making it. Take patient in dark room; dilate pupil with atropine, two grains to one ounce water; sit patient down; take lighted candle, throw light in pupil; use convex glass or ophthalmoscope. If bright reflex no cataract; light cannot pierce lens in mature cataract; lens is opaque. If cortical, may find radiating striæ; must discriminate this disease from the appearance of the pupil in glaucoma. In glaucoma, green reflex; lens also usually transparent. Turn eye from one side to the other; if cataract, same appearance; not so in glaucoma; ball not hard in cataract; hard in glaucoma; complete glaucoma, no light perceptible to patient. Never operate for any disease if no light can be detected by patient; always use dark room for examination. Take candle and sit off ten or twenty feet; ask patient to point to light; change direction of light. If can't see light at ten feet, some trouble behind lens; if can't see light at three or four feet, don't operate; if above five feet, can promise some vision; if can see light twenty feet, good vision, if no complications. Cataract usually from 40th year and upward. Congenital often; some think it due to syphilitic taint; grooved teeth an evidence of this, saw-like in appearance (not very clear to his mind).

Cataract traumatic and idiopathic; should be mature before operating; two principal modes of operating, extraction and discission; several modes of extraction; principal Von Graefe's. Preparatory treatment essential for few days; light diet; dose salts night before; don't want bowels to act for several days after operation; dilate pupil day before with atropine, two or four grains to ounce of water. Place patient on bed near edge; use speculum to hold lids open; grasp conjunctiva a line or two below cornea, with forceps; grasp on line with pupil; firm hold; depress ball; pressure not too great; hold steady hand; use Graefe's knife; pierce cornea one-third down its circumference; enter corneo-sclerotic junction, or line in front or behind; don't go back much behind cornea, or may wound ciliary bodies; inflammation of these dangerous to eye. Pass knife a little down; turn point up and make counter puncture corresponding to entry; cut up and out at corneo-sclerotic junction; always pass knife in front of iris; cut with saw-like movement. When flap is made, aqueous humour will flow out; will have collapse of cornea at times, in old and debilitated persons. Cleanse eye of secretions; make iridectomy; grasp iris at pupillary margin; draw out and clip to periphery; iridectomy made to prevent iritis, and for free passage of lens out.

Take cystotome and break up anterior capsule of lens; cut in various directions; then force lens out; press gently on lower portion of cornea; lens slips out; don't make pressure on ball with forceps; raise speculum off ball, or vitreous may escape; cortical substance may be left behind; don't leave it; get it out; may interfere with vision. During operation, vitreous may escape; a little may do good; some break up posterior capsule and let vitreous escape; if vitreous escapes before lens, raise and remove lens with spoon, or pressure will force out vitreous. After treatment, dark room; keep patient entirely still; keep pupil dilated with atropine. In making iridectomy, iris may be caught in wound of cornea; will set up iritis. Gently replace iris back with stylet; place patient in bed on back; bandage eye; no conversation; feed with spoon; don't raise up; cleanse eye each day; usually this position for five or six days; each case regulates itself. After getting up, gradually light the room; may walk about some; use dark glasses. Operation by depression obsolete; may use it in the very old; the depressed lens often sets up chronic inflammation in coats of eye, choroid and retina.

Soft variety, in young. Operation by discission. Dilate pupil and fix eye same way as for hard cataract; puncture with small needle and break up the lens; introduce the needle through sclèrotic or cornea; if passed through cornea, not over field of vision; may cause opacity where needle is introduced. Cut anterior capsule; will shrivel up; crucial incision; lens begins to swell; is permeated by aqueous humour, and absorbed; may swell too much; may set up iritis. Lens gradually breaks up; may have to repeat operation four or five times; may set up inflammation in any of these operations. One danger, inflammation of cornea; if from discission, begins in circle around puncture; grows larger and larger in cornea; cornea often destroyed. Keep pupil dilated; use ice, local blood-letting, etc. Other dangers, iritis and suppuration; slough of cornea around cut, or formation of membrane in pupillary space, result of inflammation (secondary cataract); eye may be entirely lost from inflammation. Operate at any time, summer or winter; any age, makes no difference. Some will do well, others not; statistics show ten to twenty per cent. are lost; if save ninety out of one hundred, very successful. Vision not as good as before cataract; must wear cataract glasses; have two sets, for distance and reading; No. 4 average for distance; No. 2½ for reading.

*Accumulation of Wax.*—Small glands in mucous membrane of external ear secrete wax. If exposed to cold, glands become irritated; secrete more wax when irritated; secretion semi-solid; gravitates towards drum. Patient begins to complain of deafness; may become deaf; no pain, but heavy, dull feeling. Examine well; if no wax, will see glistening appearance of membrana tympani (or drum); drum when in health looks grayish; also see bright triangular reflex; comes from drum. If wax, looks black, perfectly so; covers drum often; wax may surround a foreign body; may have been deaf for years; wax cuts off vibrations of air from drum; may become solid; often sets up no inflammation. Drum, to some degree, analogous to cornea in tissue; less sensitive. Treatment various. Don't scoop it out; dangerous; may perforate drum; use strong solution of bi-carb. soda, twenty or thirty grains to one ounce glycerine, or S. oil, or water same; fill up the ear three times daily; it breaks wax up in two or three days; then wash out with syringe; get out part at a time, or may injure drum, cause inflammation, etc. Will restore hearing at once when removed; may use forceps to pull out pieces.

*Eczema of Ear.*—Common to children; often have it; adults also; extends in meatus. Patient begins to scratch ear; keeps parts raw; if scabs are present and scratched off, clear fluid exudes; grows more solid until exudation stops, and presents a yellow, honey-comb appearance. First begins in watery pimples, with small, red base around the pimple; skin only affected; parts beneath sound; dermis only diseased; itching powerful; exudation forms scab; scab and red ring at base of pimples characteristics of disease. Only disease of that kind that forms about the ear; acute and chronic; may be chronic in meatus and last for years. Is shown by scurfy or scaly lining; this not found on outside; seldom extends to drum, but may; whole lining of membrane of external ear may come out as cast; epithelial lining of drum may also come away; may extend over face and head, etc. Treatment—At times troublesome to treat and stubborn; diachylon ointment best; ointment hard to make; not good often; it is a lead ointment, viz: linseed oil and lead plaster, equal parts; made by heating; chemical combination. Before applying, remove scabs; starch poultice best, applied at night; softens them; take off in morning and wash scabs off with tepid water and soap; afterwards take soft linen strips of cloth; spread ointment on thick as back of case knife; bind them on; apply to folds in ear with charpie. Dress once every day; water irritates disease; take off dressing and wipe parts with soft cloth; don't apply water. Physician should make application himself; do not trust to nurses; this mode will generally cure. May use borax, ten or twenty grains, to glycerine, one ounce; very good for some cases. Treatment for eczema in ear—Wipe out ear with charpie; cleanse well; apply ointment on charpie and pass in ear at night; may remove it during day; may find disease about the nose; treatment same. In chronic form, treatment about the same; may have to change application before cure is effected. If no benefit from diachylon ointment, use oleum ruscum (oil of tar); can give it pleasant flavor by adding three or four drops of lavender to ounce. Take camel's hair pencil or brush and paint it on diseased parts; continue application until skin is yellow; apply once daily. May use combination of equal parts of diachylon ointment and oil of tar, with few drops of lavender to flavor. These may fail; some use strong solution of nitrate of silver; sometimes used to destroy diseased skin, so new and healthy can form; or use borax, ten to twenty grains to water half ounce and glycerine half ounce; or alum, ten grains to ounce of glycerine or water. Oil of tar, in chronic cases, or diachylon ointment, will nearly always cure.

Diachylon ointment good for sweating feet. Bathe feet at night in warm water; it opens pores; pack charpie medicated, with ointment, between toes; keep socks on a week or so; don't wash feet. With local treatment for varieties of eczema, Fowler's Solution good; given internally.

*Myringitis* (*one of the diseases classed ear ache*).—Inflammation of membrana tympani or drum, usually; glands which secrete wax, subject to inflammation; may give rise to small pimples, filled with pus; if so, open and let it out. Drum, in health, glistening and grayish in appearance; looks like pearl; in healthy drum, bright triangular reflex; in myringitis, have pain; drum congested, red, and many small vessels shooting through it; can't see vessels in health; in centre of drum handle of malleus is visible; two vessels on each side plain in inflammation; whole drum becomes red; surrounding parts congested; intense pain in interior of ear, throbbing, itching sensation; itching becomes painful; fever often may be local or general; parts around ear hot and swollen. Press with finger on tragus, causes pain in region of drum; pain may become intense; child may become delirious; may be mistaken for brain fever; very thin plate of bone between middle ear and brain. Inflammation can extend from ear to meninges of brain through this; patient usually little deaf; due to mucous membrane of ear and eustachian tubes swelling. Myringitis sometimes an independent disease, but frequently result of irritation or inflammation of external or middle ear. Causes, cold and exposure; drum liable to inflammation; lying in window in draft of air sets up disease. Traumatic—Foreign bodies; attempting to get them out may wound drum; also bad colds; extending inflammation through eustachian tube. Treatment, simple; mastoid process behind middle ear; leeches over mastoid process, or on tragus; three or four, according to age; or cups and blisters; or apply irritating liniments, or a poultice, not immediately over ear, but around; if over, will retain hot air in the ear; this increases congestion; hop poultice best, or mustard, etc. Use solution of morphine; few grains to ounce of water; lay patient on side and fill ear up with this solution, tepid; allow it to remain five minutes. Thinks atropine to drum will do good, four or five grains to ounce of water, diluted to suit age; pour in warm; is sedative. If extracting blood, in adults, place eight or ten leeches over mastoid process, and one or two on tragus. May use constant stream of warm water to the ear for ten or fifteen minutes several times daily with good effect. Take eight or ten ounces of blood; usually almost instant relief. If pain returns, reapply leeches or blisters. May cut posterior auricular artery, if no leeches or cups; stop bleeding by compress.

